# Comparative Analysis of Chloroplast Genome Between Widely Distributed and Locally Distributed *Lysionotus* (Gesneriaceae) Related Members

**DOI:** 10.3390/ijms26157031

**Published:** 2025-07-22

**Authors:** Jia-Hui Li, Wei-Bin Xu, Chang-Hong Guo

**Affiliations:** 1 Key Laboratory of Molecular and Cytogenetics, College of Life Science and Technology, Harbin Normal University, Harbin 150025, China; hsdswlijiahui@163.com; 2 Guangxi Key Laboratory of Plant Conservation and Restoration Ecology in Karst Terrain, Guangxi Institute of Botany, Guangxi Zhuang Autonomous Region and Chinese Academy of Sciences, Guilin 541006, China

**Keywords:** *Lysionotus*, chloroplast genome, variations, genetic differences

## Abstract

The genus *Lysionotus* belongs to the family Gesneriaceae and includes plants with both ornamental and medicinal value. However, genomic-level data on the genus remains scarce. Previous investigations of *Lysionotus* have predominantly centered on morphological classification, with only limited exploration of molecular phylogenetics. Comparative analysis of chloroplast genomes within the genus would provide valuable insights into the genetic variations and evolutionary patterns of *Lysionotus* plants. In this study, we present the analysis of 24 newly sequenced chloroplast genomes from *Lysionotus*-related members, including widely distributed and locally distributed species. The results showed that the 11 plastome sizes of widely distributed species ranged from 152,928 to 153,987 bp, with GC content of 37.43–37.49%; the 13 plastome sizes of locally distributed species ranged from 153,436 to 153,916 bp, with GC content of 37.43–37.48%. A total of 24 chloroplast genomes owned typical quadripartite structures, and the number of tRNA (36 tRNAs) and rRNA (4 rRNAs) were observed for all 24 genomes. However, the number of their protein-coding sequences (CDs) varied at individual levels. No contraction and expansion of IR borders, gene rearrangements, or inversions were detected. mVISTA and Pi showed inverted repeats (IR) region was more conserved than the single copy region, coding region was more conserved than the non-coding region. Additionally, the repeat sequences and codon usage bias of *Lysionotus* plastomes were also conserved. Our results offer a comprehensive understanding of the genetic differences among these species and shed light on their phylogenetic systematics.

## 1. Introduction

Most cultivated varieties come from wild plant resources [1,2,3,4,5]. Scientific understanding and identification of wild plant resources are of great significance for domestication, cultivation, and protection of crops with important agronomic and horticultural traits [2]. Gesneriaceae plants have high ornamental value, and their wild plant resources are abundant in China [6]. These plants are mainly distributed in karst areas of southern and southwestern China, with obvious corolla tubes, gorgeous flowers, and rich colors [7]. However, only limited species are cultivated in southern China [8,9]. The Gesneriaceae family includes highly differentiated and evolved taxa, and their wild populations are very small. In particular, the endemic genera in China have the characteristic of narrow distribution [10,11]. However, some species with strong adaptability to the environment and large populations with wide distribution could also be suitable for cultivation [4,12,13,14]. The geographical distribution differences of wild resources lead to genetic variation or differentiation [15,16,17,18]. Genetic information at the genome level can be used to reveal the genetic differences of wild resources and speculate on the laws of genetic differentiation and adaptation potential of wild resources [19]. Therefore, more efforts should be invested in introducing, domesticating and commercializing the widely distributed species with strong adaptability [4,20,21]. Moreover, protecting species adapted to unique habitats from extinction is an important basis for the protection of wild resources and ecological balance [20].

Chloroplasts are usually non-recombinant and uniparentally inherited organelles that convert light energy into chemical energy in plants [22,23]. Since the discovery of the chloroplast genome, it has been widely used in the study of plant systematics, identification and protection of plant germplasm resources, regulation mechanism of photosynthesis, evaluation of plant genetic diversity, detection of interspecific hybridization of plants, and research on plant genetic engineering [24,25]. With the development of gene sequencing technology, a large number of plant chloroplast genome sequences have been published. However, species with high economic, medicinal, and edible values have not been widely sequenced yet [26,27]. Chloroplast DNA (cpDNA) has been increasingly employed for resolving the deep phylogeny of plants because of their low rates of nucleotide substitutions and decelerated structural variation compared with nuclear genomic sequences [28,29,30]. Based on this, comparing chloroplast genomes of wild plants distributed in different regions could predict the genetic variation basis of wild germplasm adaptation to different habitats, helping in understanding the genetic adaptation mechanism.

*Lysionotus* is a typical dominant genus of Gesneriaceae in China [31], with about 37 species. Most species are shrubs or subshrubs, distributed in northern India and Nepal, eastward through Bhutan, Laos, Myanmar, Thailand, northern Vietnam, and southern China to southern Japan [32,33]. The only two centers of diversity in this genus are the Guangxi–Guizhu–Yunnan karst region and Yunnan–southwest Tibet and northeast India [34]. With two diversity centers, China harbors the highest diversity of the genus, with at least 25 species and six varieties [35,36]. Based on the phylogenetic relationships obtained from previous studies and the experience of field scientific investigation, this genus has two genetically related evolutionary clades with opposite geographic distribution patterns [37]. The first clade includes *Lysionotus pauciflorus*, *Lysionotus pauciflorus* var. *ikedae*, *Lysionotus aeschynanthoides*, *Lysionotus pterocaulis*, *Lysionotus wilsonii*, *Lysionotus microphyllus* var. *omeiensis*, *Lysionotus kwangsiensis*, *Lysionotus heterophyllus*, and *heterophyllus* var. *mollis*. These species generally possess strong adaptability to habitats and wide distribution. The second clade contains *Lysionotus pubescens*, *Lysionotus atropurpureus*, *Lysionotus sessilifolius*, *Lysionotus forrestii*, *Lysionotus levipes*, *Lysionotus chatungii*, *Lysionotus petelotii*, *Lysionotus sulphureoides*, *Lysionotus serratus*, *Lysionotus metuoensis* and *Lysionotus gamosepalus*, which mainly grow in the unique geographical environment of Yunnan and Tibet, and are mostly epiphytic. Therefore, *Lysionotus* species are excellent materials for studying adaptive differentiation among related species. Comparative analysis of chloroplast genomes between the widely distributed clade and the locally distributed clade could reveal genetic differences between these two major evolutionary lineages. Moreover, based on chloroplast genomes, a more comprehensive phylogenetic framework can be constructed, providing insights and examples for molecular systematics, genetic diversity, conservation, and even speciation processes in related plant groups.

A previous study sequenced and characterized the chloroplast genome of *L. pauciflorus*, providing initial insights into its structure, gene content, and organization [38], and highlighting the potential of chloroplast genomic data for conservation genetics in Gesneriaceae. However, as this work focused on a single species, comprehensive comparative data across *Lysionotus*—especially among species with varied geographic distributions and ecological adaptations—remain lacking.

To date, no systematic comparative analysis of chloroplast genomes among multiple *Lysionotus* species has been performed. Such studies are essential to elucidate genetic mechanisms of adaptation, evolutionary divergence, and phylogenetic relationships within the genus, as well as to identify hypervariable regions and repeats useful for developing molecular markers for breeding and conservation.

In this study, we extend the work of Ren et al. by sequencing and analyzing the chloroplast genomes of 24 representative *Lysionotus* species from two genetically distinct clades with contrasting distribution patterns. Through comparative genomics and phylogenetic analyses, we aim to uncover genetic differentiation between widely and locally distributed species, elucidate evolutionary mechanisms, and provide molecular resources to support biodiversity conservation and breeding programs in this genus. With the exception of *L. pauciflorus*, the chloroplast genomes of the other species are reported here for the first time. Our main contributions are as follows: (1) we obtained and assembled the chloroplast genomes for all 24 samples; (2) we compared the structural characteristics and sequence divergence of the chloroplast genomes within the two *Lysionotus* clades; (3) we identified hypervariable regions between the widely distributed and locally distributed *Lysionotus* species; (4) we counted and compared simple sequence repeats (SSRs) and large repeat sequences (LRSs) in the widely distributed and locally distributed clades; and (5) we inferred the phylogenetic relationships of the two clades based on common single-copy coding sequences from the chloroplast genomes.

## 2. Results

### 2.1. Features of the Plastome Genome

A total of 24 chloroplast genomes were studied, which belong to two genetically similar evolutionary clades. The large single copy (LSC) region had the greatest variation in length, mainly causing length variation of the chloroplast genome. No large fragment deletions were detected in the inverted repeats (IR) region, and the lengths of two IR regions were consistent in all chloroplast genomes. The GC content of each species was highly similar throughout the entire chloroplast genome, and the same was true for the same regions (LSC, IR, and small single copy, i.e., SSC), though the GC content in IR regions was higher than in other regions.

There were 11 datasets in a widely distributed clade, including *L. pauciflorus* from Fujian (*L. pauciflorus* FJ) and Yunnan (*L. pauciflorus* YN), *L. pauciflorus* var. *ikedae*, *L. aeschynanthoides*, *L. pterocaulis*, *L. heterophyllus* from Guizhou (*L. heterophyllus* GZ) and Guangxi (*L. heterophyllus* GX), *L. wilsonii*, *L. microphyllus* var. *omeiensis*, *L. kwangsiensis* and *L. heterophyllus* var. *mollis* (Table 1). Chloroplast genome sizes in this clade ranged from 152,928 to 153,987 bp (*L. pauciflorus*). GC content ranged from 37.43% (*L. heterophyllus* var. *mollis*) to 37.49% (*L. microphyllus* var. *omeiensis*). LSC length ranged between 84,077 and 85,131 bp, where the smallest was *L. pterocaulis* and the longest was *L. pauciflorus* YN. SSC length ranged from 17,746 to 17,922 bp; the smallest was *L. kwangsiensis* and the longest was *L. heterophyllus* GX. IR length was in the range of 25,461–25,485 bp. The smallest IR was recorded for *L. pauciflorus* var. *ikedae*, while *L. pauciflorus* YN depicted the longest IR.

There were 13 datasets in the locally distributed clade. Species in this clade included *L. gamosepalus*, *L. pubescens*, *L. atropurpureus*, *L. sessilifolius*, *L. forrestii*, *L. levipes*, *L. chatungii*, *L. guiliangii*, *L.* sp., *L. petelotii*, *L. sulphureoides*, *L. serratus*, and *L. metuoensis*. Chloroplast genome sizes in this clade ranged from 153,436 to 153,916 bp, with *L. atropurpureus* having the shortest genome and *L. forrestii* possessing the longest. GC content ranged from 37.43% to 37.48%, with *L. guiliangii* possessing the shortest GC content and *L.* sp. having the longest. LSC length varied from 84,592 to 85,069 bp. *L. atropurpureus* showed the shortest LSC and the longest was observed in *L. forrestii*. SSC length ranged from 17,780 to 17,896 bp, the shortest was *L. guiliangii,* and the longest was *L. atropurpureus*. IR length was observed within the range of 25,473 to 25,488 bp. *L. pubescens* had the shortest IR and the longest was observed in *L. gamosepalus* (Table 2).

### 2.2. Genome Annotation

**Figure 1:**
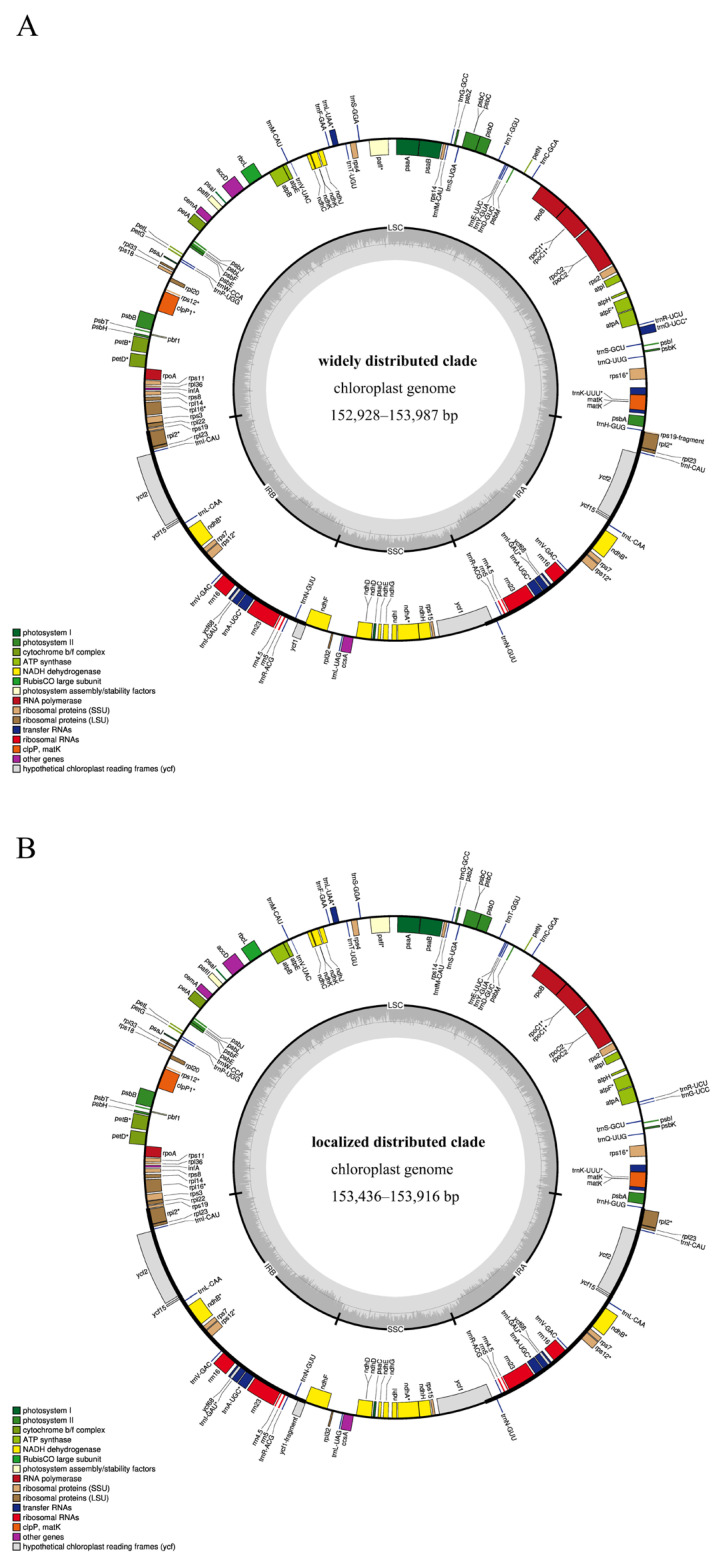
Structure of assembled and annotated chloroplast genomes. The genes outside the circle were transcribed counterclockwise, whereas those inside the circle were transcribed clockwise. The gene content and organization were similar for all species. (**A**) represents the widely distributed clade species, while (**B**) represents locally distributed clade species; * indicates that the gene contains an intron.

All chloroplast genomes owned typical quadripartite structures (Figure 1). A constant number of transfer RNA (36 tRNAs) and ribosomal RNA (4 rRNAs) were observed for all 24 genomes. However, the number of their protein coding sequences (CDs) varied at individual levels, but were similar between the two clades (Table S1).

The widely distributed clade, except for *L. heterophyllus* GX possessing 79 CDs, exhibited the most prominent variation compared to the others, with the remaining 10 chloroplast genomes all containing 80 CDs. In the locally distributed clade, *L. sulphureoides* showed 79 CDs, while the remaining 12 chloroplast genomes possessed 80 CDs (Table 2).

### 2.3. Genomic Divergence

The IR region displayed a significantly higher degree of sequence conservation in comparison to the single copy region (Figure S1). In contrast, the coding regions exhibited more pronounced evolutionary conservatism compared with the non-coding regions. The distribution of cyan was primarily observed in two reversed repeats within the chloroplast genome. The trends remained consistent across the two regions. The curve exhibited a high peak, and it was positioned closely to the horizontal axis, indicating a diminished level of genetic diversity, consistent similarity, and uniform genetic structure between two reverse repeat regions of 24 chloroplast genomes. The main variation regions included intergenic region spacer (IGS) and CDs, IGS (*rps16-trnQ-UUG*), IGS (*rpoB-trnC-GCA-petN-psbM-trnD-GUC-trnY-GUA-trnE-UUC-trnT-GGU-psbD*), IGS (*ycf3-trnS-GGA-rps4-trnT-UGU-trnL-UAA-trnF-GAA-ndhJ*), IGS (*ycf4-cemA*), IGS (*rpl16*), IGS (*rps12-rpl32-trnL-UAG-ccsA-ndhD-psaC-ndhE-trnR-UCU-ndhG-ndhI*) and CDs (*ycf1*).

**Figure 2:**
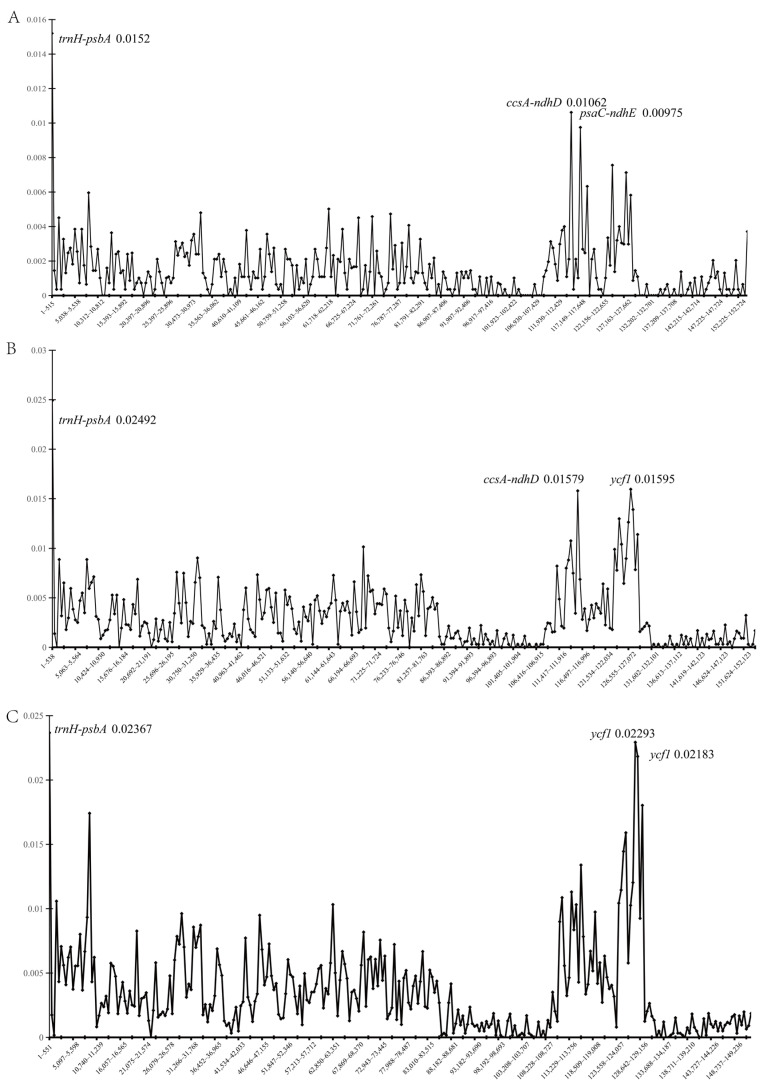
Nucleotide diversity (Pi) is based on sliding window analysis in aligned complete chloroplast genomes. (**A**) the chloroplast genome Pi of 11 widely distributed species, (**B**) the chloroplast genome Pi of 13 locally distributed species, (**C**) the Pi of 24 chloroplast genomes.

Nucleotide diversity index Pi was used to quantify genetic diversity (Figure 2). The region with the highest average Pi in the widely distributed clade (11 chloroplast genomes) was *trnH-psbA* (Pi = 0.0152), followed by *ccsA-ndhD* (Pi = 0.01062) and *psaC-ndhE* (Pi = 0.00975). In the localized distributed clade (13 chloroplast genomes), *trnH-psbA* (Pi = 0.02492), *ycf1* (Pi = 0.01595) and *ccsA-ndhD* (Pi = 0.01579) had high Pi. The highly variable regions of both clades contained *trnH-psbA* and *ccsA-ndhD*. However, the localized clade exhibited higher genetic diversity and greater sequence differences. The Pi revealed troughs primarily occurring between 82,291–111,930 and 127,662–147,225 in the widely distributed clade, exhibiting low genetic diversity (Figure 2A). The region with low genetic diversity of the localized distributed clade was in the range of 86,393–106,915 and 131,602–147,123 (Figure 2B). The low variation for all 24 chloroplast genomes ranged between 88,681–108,727 and 129,156–149,236 (Figure 2C). These regions were generally inverted repeat regions, consistent with mVISTA results. The Pi of all samples put together was also calculated, with no significant change in the high variable region. In summary, coding regions (except *ycf1*) were more conservative, with lower Pi values. Non-coding regions usually had higher variation, with higher Pi values.

### 2.4. Contraction and Expansion Analysis of IR Region

The typical quadripartite structure of chloroplast genome results in four boundaries: LSC/IRb (JLB line), IRb/SSC (JSB line), SSC/IRa (JSA line), and IRa/LSC (JLA line). The contraction/expansion of IR region caused evolution and variations in chloroplast GC content and genome size of plant. IR region variation was related to species’ relationship, geographical distribution and ecological adaptation. To better understand the impact of gene transfer or indel events, both within and outside the IR region, we examined the chloroplast genome features of 24 samples and evaluated the phenomenon of IR contraction and expansion.

**Figure 3:**
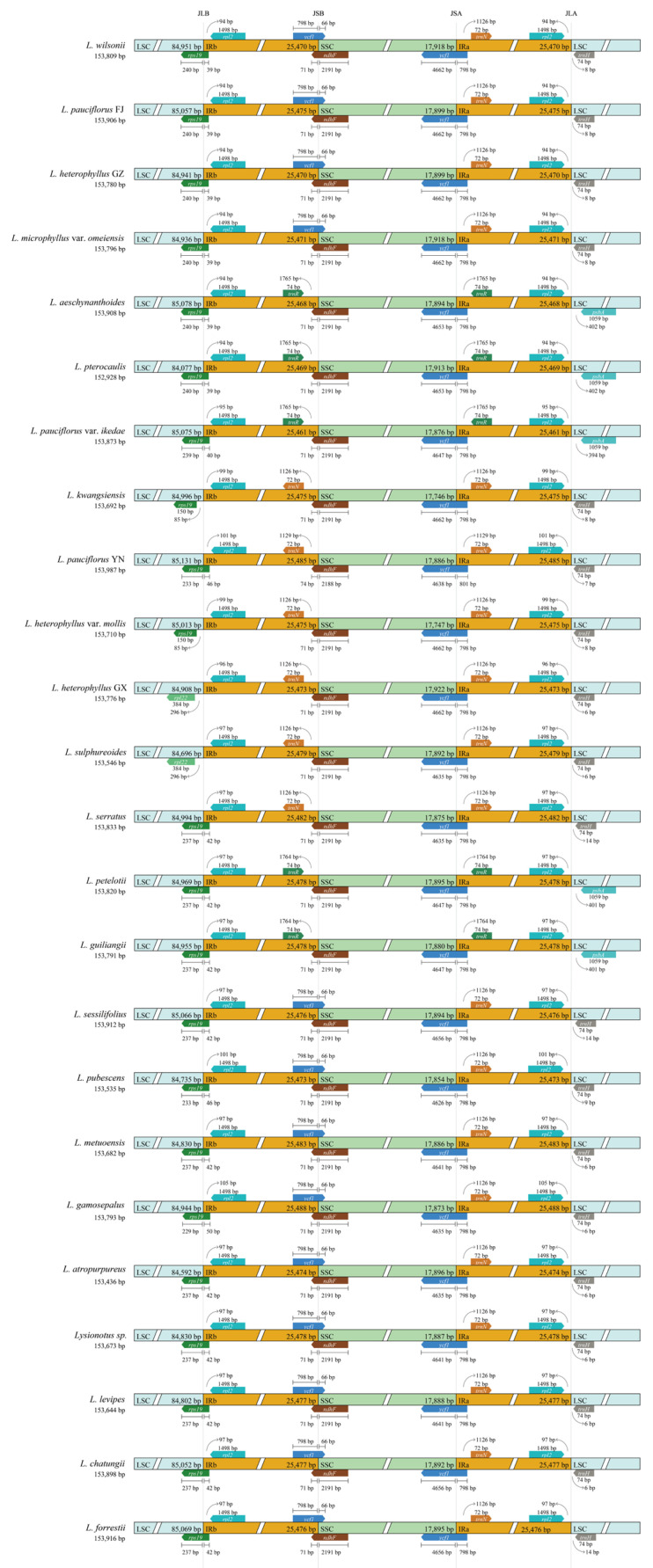
Details of the contraction and expansion of inverted repeats at junction sites. For all plants, positive strand genes were represented at the top, from right to left, on the corresponding track, whereas negative strand genes were illustrated on the lower side of the track, from left to right. JLB, JSB, JSA and JLA represent the border loci of LSC/IRb, IRb/SSC, SSC/IRa and IRa/LSC, respectively.

According to CPJSdraw, arrows depict the distance between the start and end of a gene from the junction site. The scale bar on the upper or lower parts of the genes extending from one region to another illustrates the number of base pairs to which genes are connected in that specific region. The *rps19*, *ndhF*, *ycf1*, and *trnH* genes were located at LSC/IRb, IRb/SSC, SSC/IRa, and IRa/LSC regions, respectively (Figure 3) in most of the chloroplast genomes. The *rps19* gene was detected in 22 chloroplast genomes, and *L. kwangsiensis* and *L. heterophyllus* var. *mollis* were completely located in the LSC region. *L. heterophyllus* GX in the widely distributed clade, and *L. sulphureoides* in the locally distributed clade, missed the *rps19* gene, which was replaced by the *rpl22* gene located in the LSC region with 296 bp apart from JLB line.

Excluding *L. heterophyllus* GX, *L. kwangsiensis* and *L. heterophyllus* var. *mollis* in the widely distributed clade, the *rps19* gene in another 8 chloroplast genomes spanned between the LSC and IRb regions, and the distances between *rps19* and JLB line ranged from 233 to 240 bp. The *ndhF* gene was located in the SSC region, with a distance of 2188–2191 bp from the JSB line (boundary between IRb and SSC). In addition, *L. wilsonii*, *L. pauciflorus* FJ, *L. heterophyllus* GZ, and *L. microphyllus* var. *omeiensis* had *ycf1* pseudogenes in IRb and also contained 72 bp *trnN* gene in IRa region. *L. aeschynanthoides*, *L. aeschynanthoides* and *L. pauciflorus* var. *ikedae* each conained 74 bp *trnR* gene in IRb and IRa regions. *L. kwangsiensis*, *L. pauciflorus* YN, *L. heterophyllus* var. *mollis* and *L. heterophyllus* GX each exhibited 72 bp *trnN* gene in IRb and IRa regions.

Except for *L. sulphureoides* in the locally distributed clade, the *rps19* gene in 12 chloroplast genomes was located on LSC and IRb regions, and the distances between *rps19* and JLB line ranged from 229–237 bp. The distance of *ndhF* in the SSC region to JSB line was 2191 bp for 13 chloroplast genomes, which were more conserved compared with the widely distributed clade. Most of the species in the localized clade, such as *L. sessilifolius*, *L. pubescens*, *L. metuoensis*, *L. gamosepalus*, *L. atropurpureus*, *L.* sp., *L. levipes*, *L. chatungii* and *L. forrestii*, had *ycf1* pseudogene in IRb region and 72 bp *trnN* gene in IRa region at the same time. *L. petelotii* and *L guiliangii* had 74 bp *trnR* gene in IRb and IRa regions. *L. sulphureoides* and *L. serratus* had 72 bp *trnN* gene in IRb and IRa regions. The length of *trnR* and *trnN* genes was highly conserved both the clades.

*TrnH* served as the boundary between the IRa and SSC regions, which was consistent with previous studies. *L. aeschynanthoides*, *L. pterocaulis* and *L. pauciflorus* var. *ikedae* in the widely distributed clade lost *trnH* gene (these three species had 74 bpd *trnR* gene in IRb and IRa regions, respectively). Similarly, *trnH* genes were also lost in *L. petelotii* and *L. guiliangii* in the locally distributed clade, while *trnH* in other samples were located in LSC region with 74 bp distance away from the JLA line. In summary, chloroplast genome evolution in the widely distributed clade and the locally distributed clade was relatively conservative. The *ndhF* was a relatively stable boundary gene with a broader distance range from JSB line in the widely distributed clade (2188–2191 bp). Samples in the localized distributed clade had a similar structure because the *ndhF* had the same distance from JSB (2191 bp). The variation range of boundary gene length in the widely distributed clade and the diversity of genes on two sides of the boundary provided more possibilities for IR boundary expansion/contraction.

### 2.5. Repeat Analysis

**Figure 4:**
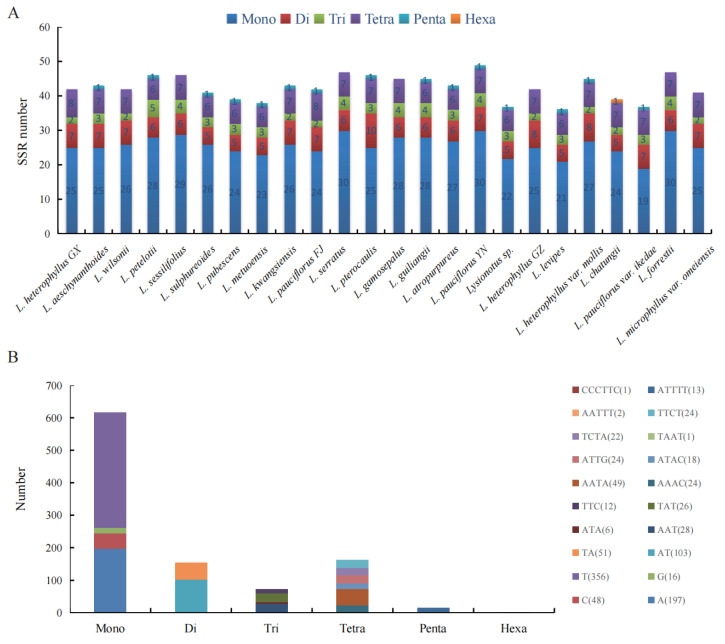
Analysis of simple sequence repeats (SSRs) in chloroplast genomes. (**A**) Statistics of different SSR types detected in each species. (**B**) Type and frequency of each identified SSR.

We compared the SSRs of 24 chloroplast genomes to understand overall distribution, types, and the number of highly similar repeats in widely and locally distributed clades (Table S2). The number of SSRs identified in the 24 chloroplast genomes ranged from 36 to 49 (Figure 4A). Mononucleotides, dinucleotides, trinucleotides and tetranucleotides were prevalent in 24 chloroplast genomes. A total of four types of mononucleotides were detected, with a large difference in number. The most abundant mononucleotides were T (356 polythymine), followed by A (197 polyadenine), C (48 polycytosine), and G (16 polyguanine). A total of two types of dinucleotides were detected, including 103 ATs and 51 TAs. A total of four types of trinucleotides were detected, including AAT, ATA, TAT and TTC, with 28, 6, 26 and 12 repeat numbers, respectively. Seven types of tetranucleotides included AATA, AAAC, ATAC, ATTG, TAAT, TCTA and TTCT, with repeat numbers of 49, 24, 18, 24, 1, 22 and 24, respectively. Pentanucleotides were present in *L. aeschynanthoides*, *L. petelotii*, *L. sulphureoides*, *L. pubescens*, *L. metuoensis*, *L. kwangsiensis*, *L. pauciflorus* FJ, *L. pterocaulis*, *L. guiliangii*, *L. atropurpureus*, *L. pauciflorus* YN, *L.* sp. MT, *L. levipes*, *L. heterophyllus* var. *Mollis* and *L. pauciflorus* var. *ikedae.* ATTTT and AATTT were two different types of pentanucleotides, with repeat numbers of 13 and 2, respectively. Only one hexanucleotide repeat CCCTTC was detected, and it existed in *L. chatungii* (Figure 4B).

**Figure 5:**
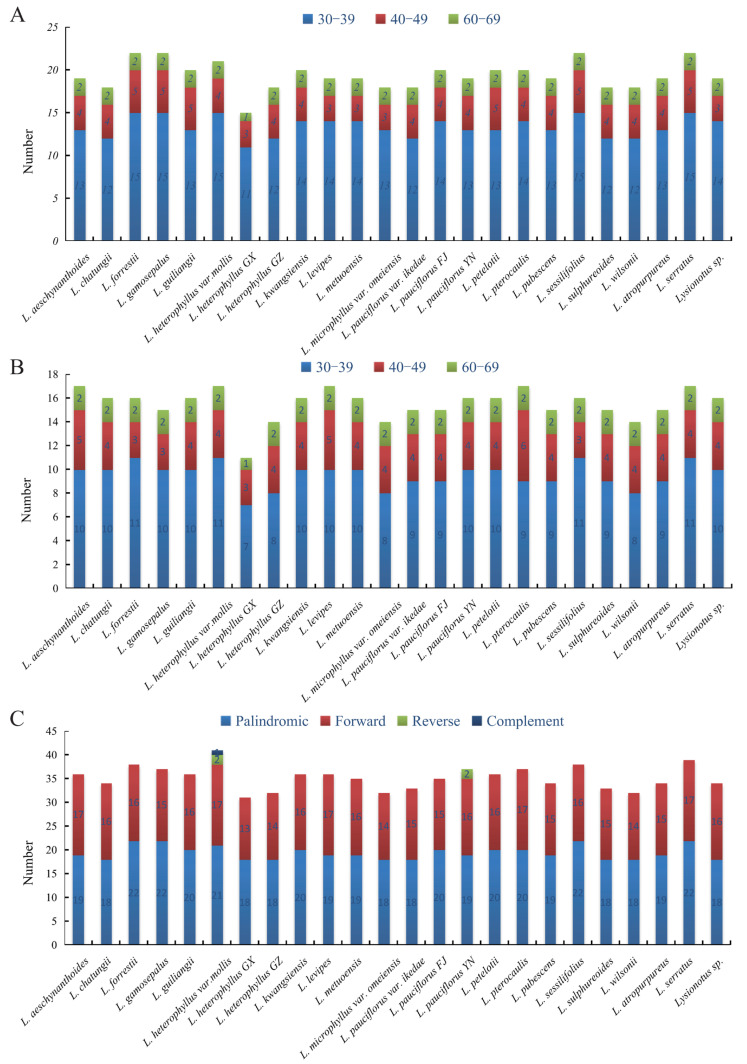
Analysis of large sequence repeats in chloroplast genomes. (**A**) Frequency of forward repeats. (**B**) Frequency of palindromic repeats. (**C**) Statistics of four types detected in each sample.

We detected 32–41 pairs of LRSs, greater than 30 bp, consisting of forward repeats (F), reverse repeats (R), complementary repeats (C), and palindromic repeats (P) in 24 *Lysionotus* chloroplast genomes (Table S3). The LRSs number of the widely distributed clade ranged from 32 to 41 bp, whereas the number of the localized distributed clade ranged from 33 to 39 bp. Among these LRSs, the number of forward and palindrome repeats were considerably higher than reverse and complement repeats. Except for *L. heterophyllus* var. *mollis* (four LRSs, P, F, R, and C) and *L. pauciflorus* YN (three LRSs, P, F, and R), the other 22 chloroplast genomes only contained two LRSs, i.e., P and F. The number of forward repeat lengths per interval (30–39 bp, 40–49 bp and 60–69 bp) were shown in Figure 5A.

The number of palindromic repeats in 24 chloroplast genomes was higher than forward repeats, and the length was mainly 30–39 bp. Figure 5B shows the number of palindromic repeats in each chloroplast genome, with sizes being set in three intervals: 30–39 bp, 40–49 bp and 60–69 bp. The palindromic repeats number of 24 chloroplast genomes among 30–39 bp ranged from 11–15 bp. The number of palindromic repeats for the widely distributed clade ranged from 11 to 15 bp, while it was 12–15 bp for the localized distributed clade. The number of forward repeats of each chloroplast genome in 30–39 bp interval was at least 7 F and 11 F at most (Figure 5C). The number in the widely distributed clade was in the range of 7–11 bp, and the number in locally distributed clade ranged from 9 to 11. In summary, the widely distributed clade dominated a broader range of palindromic repeat numbers (11–15) and forward repeat numbers (7–11) in the 30–39 bp interval (versus the localized distributed clade: 12–15 P, 9–11 F). Furthermore, *L. pauciflorus* YN and *L. heterophyllus* var. *mollis* also belonged to the widely distributed clade and possessed more abundant types of LRSs, suggesting that the widely distributed clade may possess more special structures or functions, such as potential adaptability.

### 2.6. Codon Usage Bias Pattern

**Figure 6:**
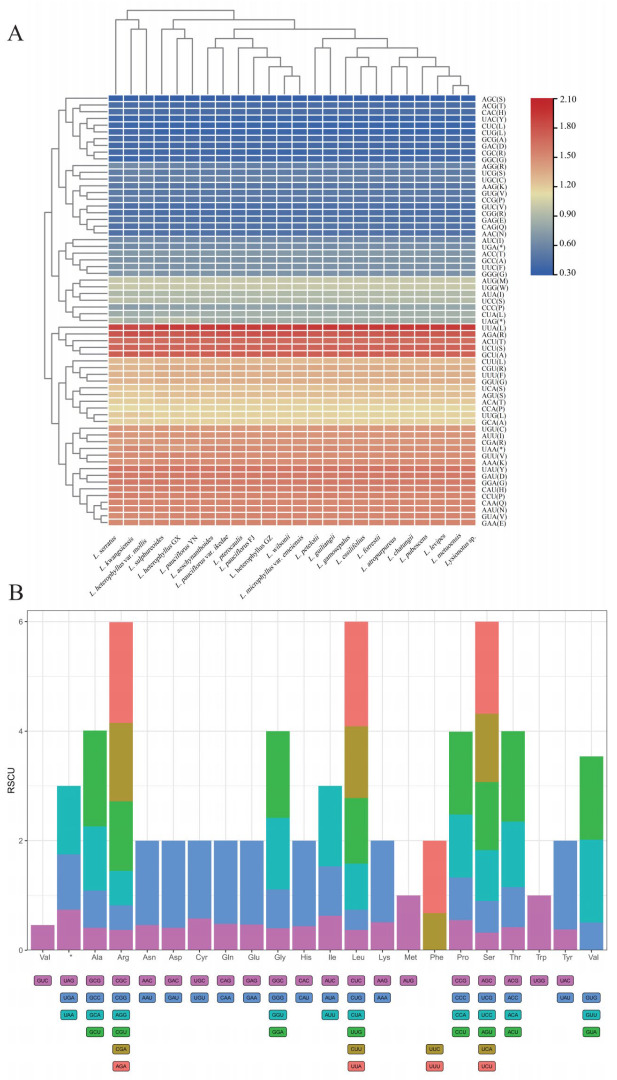
The RSCU of 89 protein-coding sequences represent 24 samples. (**A**) The heatmap of RSCU of 64 codons across 24 chloroplast genomes. Red boxes imply highly preferred synonymous codons (RSCU > 1), blue boxes indicate less preferred synonymous codons (RSCU < 1). (**B**) The average RSCU of 24 chloroplast genomes. Each column is divided into different colored segments, each color representing one type of codon. The height of the column indicates the total RSCU of amino acid. The height of the different colors indicates RSCU of corresponding codon.

The number of codons encoding the same amino acid was more conserved across *Lysionotus* species. *L. atropurpureus* in the locally distributed clade exhibited a higher frequency compared to codons in other species. However, the number of codons encoding different amino acids varied greatly. Among 64 codons in 24 chloroplast genomes, there were 30 more preferred codons (relative synonymous codon usage, ie., RSCU > 1), two equally preferred codons (RSCU = 1) (UGG and AUG), and 32 less preferred codons (RSCU < 1) (Figure 6A). In the widely distributed clade, AUA encoding isoleucine (Ile) was the most abundant (1095 in *L. kwangsiensis*), while the UGU encoding cysteine (Cys) was the least (80 in both *L. aeschynanthoides* and *L. pterocaulis*). Additionally, there were 135,446 codons ended with A/U and 140,586 codons ended with G/C. In the locally distributed clade, UAU encoding tyrosine (Tyr) was the most abundant (2092 in *L. atropurpureus*), and GCA encoding alanine (Ala) showed the least abundance (78 in *L. petelotii*, *L. sulphureoides* and *L. guiliangii*). Additionally, there were 172,087 codons ended with A/U and 178,439 codons ended with G/C (Table S4). Based on RSCU and observed frequency, the codon usage was biased for G/C endings in two different clades.

The RSCU of 64 codons (including UAA, UAG and UGA stop codons) encoding amino acids are shown in Figure 6B, where each column represents an amino acid. Leucine (Leu), arginine (Arg) and serine (Ser) featured the highest total RSCU (RSCU = 6), these amino acids were biased to use UUG, UUA and CUU for leucine (Leu); CGU, CGA and AGA for arginine (Arg); and UCU, UCA and AGU for serine (Ser). Followed by alanine (Ala), glycine (Gly), proline (Pro), threonine (Thr) and valine (Val), these amino acids featured a total RSCU of 4. Alanine (Ala) was biased to use GCU and GCA, glycine (Gly) was biased to use GGU and GGA, and proline (Pro) was biased to use CCU and CCA. Threonine (Thr) was biased to use ACU and ACA, and valine (Val) was biased to use GUU and GUA. Moreover, asparagine (Asn) exhibited a preference for the AAU codon, the GAU codon was inclined towards aspartate (Asp), cysteine (Cys) was biased to use UGU, glutamine (Gln) showed tendency towards CAA, glutamic acid (Glu) was biased to use GAA, histidine (His) was inclined towards CAU, lysine (Lys) preferred to use AAA, UUU was employed by phenylalanine (Phe) and tyrosine (Tyr) was biased to use UAU. Isoleucine (Ile) was encoded by three different codons, but only AUU was greater than 1 (RSCU = 1.47). Methionine (Met) and tryptophan (Trp) were encoded by AUG and UGG, and these two codons were equally preferred codons (RSCU = 1).

### 2.7. Phylogenetic Relationships

**Figure 7:**
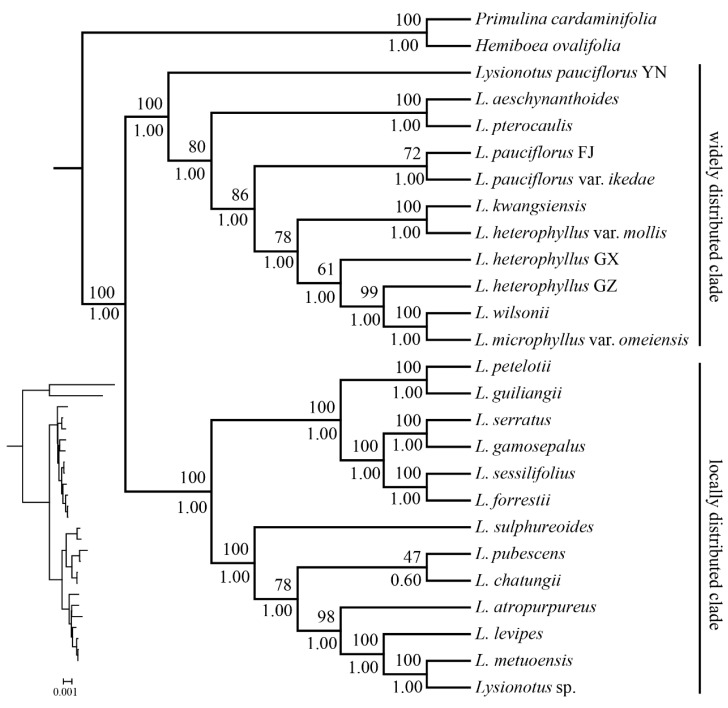
Phylogenetic analysis of 24 chloroplast genomes based on ML and BI joint methods.

**Figure 8:**
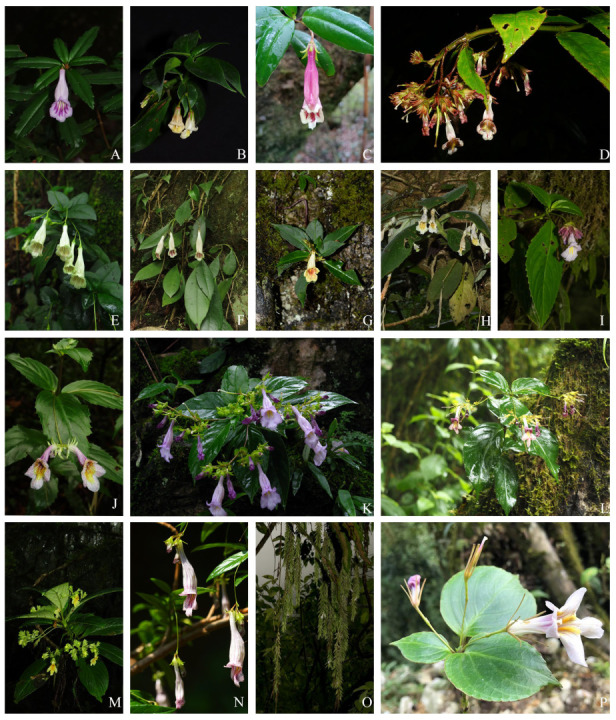
Morphological characteristics of *Lysionotus* species in two distributional clades (widely distributed: (**A**–**F**); locally distributed: (**G**–**P**)). (**A**). *L. pauciflorus* FJ; (**B**). *L. aeschynanthoides*; (**C**). *L. kwangsiensis*; (**D**). *L. pterocaulis*; (**E**). *L. heterophyllus* GX; (**F**). *L. wilsonii*; (**G**). *L. petelotii*; (**H**). *L. guiliangii*; (**I**). *L. gamosepalus*; (**J**). *L. sessilifolius*; (**K**). *L. serratus*; (**L**). *L. atropurpureus*; (**M**). *L. sulphureoides*; (**N**). *L. pubescens*; (**O**). *L. metuoensis*; (**P**). *L. chatungii*.

Choosing *Hemiboea ovalifolia* and *Primulina cardaminifolia* as outgroups, 24 samples in this study formed two clades; the phylogenetic relationship is shown in Figure 7. The support rates of ML and posterior probabilities of BI trees were marked above and below the branches in joint support tree. The morphological characteristics of some representative species are shown in Figure 8, where panels A–F depict species in the widely distributed clade (e.g., *L*. *pauciflorus* and *L. heterophyllus*), and panels G–P illustrate species in the locally distributed clade (e.g., *L. atropurpureus* and *L. sessilifolius*). In the first widely distributed clade, *L*. *pauciflorus* from Yunnan separated earliest, followed by *L. aeschynanthoides* and *L. pterocaulis*, which further clustered together. The *L. pauciflorus* from Fujian and *L. pauciflorus* var. *ikedae* further clustered together. *L*. *kwangsiensis* and *L. heterophyllus* var. *mollis*, both from Guangxi, diverged at a relatively intermediate stage and exhibited a closer phylogenetic relationship within their clade. In contrast, *L. heterophyllus* from Guizhou and Guangxi, *L. wilsonii* from Sichuan, and *L. microphyllus* var. *omeiensis* from Chongqing, diverged most recently, as shown by their terminal positions in the phylogenetic tree.

Species in the second locally distributed clade could further form two groups, the first group including six species: *L. petelotii*, *L. guiliangii*, *L. serratus*, *L. gamosepalus* from, *L. sessilifolius* and *L. forrestii.* The second group includes seven species: *L. sulphureoides* and *L. pubescens*, *L. sulphureoides*, *L. atropurpureus*, *L. levipes* and *L.* sp. Despite the geographical proximity of Sichuan and Tibet, the phylogenetic relationship of species from the two regions was relatively distant, and they evolved relatively independently in different clades. Moreover, although species *L. gamosepalus*, *L. atropurpureus*, *L. metuoensis* and *Lysionotus* sp. were both from Motuo, *L. gamosepalus* was more closely related to *L. serratus* from Guangxi, while *L. atropurpureus* possibly experienced a more complex evolution, and underwent adaptive evolution in the Tibet.

The species in the second local clade can be divided into two groups. The first group consists of six species: *L. petelotii*, *L. guiliangii*, *L. serratus*, *L. gamosepalus*, *L. sessilifolius*, and *L. forrestii.* The second group includes seven species: *L. sulphureoides*, *L. pubescens*, *L. atropurpureus*, *L. levipes*, and an unidentified species of *L*. sp. Despite the geographical proximity of Sichuan and Tibet, the phylogenetic relationships among species from these two regions are relatively distant, indicating that they have evolved independently in different clades. Furthermore, although *L. gamosepalus*, *L. atropurpureus*, *L. metuoensis*, and *L*. sp. all originate from Motuo, *L. gamosepalus* is more closely related to *L. serratus* from Guangxi. In contrast, *L. atropurpureus* appears to have undergone a more complex evolutionary process, possibly undergoing adaptive evolution in Tibet. This analysis suggests that, despite their proximity, the species from different regions may exhibit significant phylogenetic divergence due to differences in their environments, ecologies, and evolutionary histories.

### 2.8. Positive Selection Based on Chloroplast

Among the 78 single-copy CDS genes initially considered for positive selection analysis (Table S1), 69 genes were ultimately selected (Table 3). Among all the genes, only the *rbcL* gene was detected to be under significant positive selection (*p* = 8.77 × 10^−4^). No significant positive selection was detected in other genes (*p*-value > 0.05). This may indicate that the *rbcL* gene has experienced special selective pressure during the evolutionary process of the species. This may be because the biological functions in which it is involved, such as those related to photosynthesis, play an important role in the species’ adaptability to the current environment. As a result, it has been favored by natural selection, and its gene frequency may have changed significantly in the population to adapt to the environment.

However, in the Bayesian Empirical Bayes (BEB) test, codon sites in 10 genes were found to have high posterior probabilities (*atpA*, *cemA*, *clpP*, *ndhA*, *ndhB*, *ndhC*, *ndhE*, *ndhH*, *rps8*, and *ycf1*) (Table 3). Previous studies have shown that codon sites with high posterior probabilities should be considered as positively selected sites [39], implying that these 10 genes might also be under positive selection pressure [38]. They have undergone adaptive changes during the differentiation of the widely distributed clade and the locally distributed clade in *Lysionotus*, although such changes did not reach statistical significance in traditional positive selection tests based on *p*-values.

## 3. Discussion

### 3.1. Chloroplast Genome Structure and Evolutionary Conservation

This study presents the first comprehensive comparative analysis of chloroplast genomes across 24 *Lysionotus* species, revealing that all genomes possess the canonical quadripartite structure, consistent with the single-species analysis of *L. pauciflorus* reported by Ren et al. [40]. Notably, the variation in genome length between the widely distributed and locally distributed clades is primarily attributed to differences in the large single-copy (LSC) region, rather than expansions or contractions of the inverted repeat (IR) regions. This pattern contrasts with that observed in many angiosperms, where IR variation typically drives genome size changes [29]. The remarkable conservation of IR regions is likely linked to their inclusion of rRNA gene clusters, which are essential for ribosomal function, as further supported by their elevated GC content (43.14–43.19%). Furthermore, the absence of gene rearrangements or large fragment deletions underscores the exceptional structural stability of *Lysionotus* chloroplast genomes, contrasting sharply with the more dynamic plastomes observed in other Gesneriaceae genera such as *Henckelia* [32,41].

### 3.2. Hypervariable Regions and Ecological Adaptation

Through mVISTA alignment and nucleotide diversity analyses, we identified *trnH-psbA* and *ccsA-ndhD* as shared hypervariable regions across both clades, while the *ycf1* gene exhibited notably higher divergence within the locally distributed species. These findings reinforce the utility of ycf1 as an effective species-discriminating marker, as previously suggested by Shaw et al. [28]. The elevated genetic diversity observed in locally distributed clades, exemplified by *L. atropurpureus*, likely reflects adaptation to the heterogeneous microhabitats of the Himalayan region. In contrast, the relatively low variation in widely distributed species such as *L. pauciflorus* suggests a more generalized environmental adaptation. Interestingly, non-coding regions exhibited greater variability than coding regions, consistent with relaxed selective constraints on intergenic spacers. The *trnH-psbA* region, involved in photosystem II assembly, may underpin adaptive differentiation in light-harvesting efficiency, whereas the potential role of *ccsA-ndhD* (implicated in cytochrome c biosynthesis) variations in high-altitude adaptation warrants further functional validation [19].

### 3.3. Phylogenetic Insights: Comparison with Previous Studies

Previous phylogenetic trees of *Lysionotus* based on nuclear *ITS* and chloroplast *trnL-F* sequences resolved two major clades but showed limited resolution within clades [37]. In contrast, our chloroplast genome-based phylogenetic analysis (Figure 7) provides higher resolution, and exhibits overall topological congruence with the phylogeny constructed using 649 orthologous genes [42]. Only a few species show discordant phylogenetic relationships. For example, *L. atropurpureus* clusters with *L. levipes* in the chloroplast tree, whereas it diverges earliest as a basal lineage in the nuclear gene tree, being closely related to *L. serratus* and *L. gamosepalus*. This discrepancy may be attributed to incomplete lineage sorting or historical hybridization events.

In this study, the early divergence of *L. pauciflorus* from Yunnan and the close clustering of Fujian populations with *L. pauciflorus* var. *ikedae* likely resulted from geographic isolation driven by enhanced East Asian monsoons during the Miocene. The Yunnan population evolved independently due to the topographic barrier of the Hengduan Mountains, while Fujian populations dispersed to Japan via land bridges during the Pleistocene, forming the variety. Notably, *L. gamosepalus* from Medog, Tibet, clusters with *L. serratus* from Guangxi rather than sympatric *L. atropurpureus*, implying long-distance dispersal during the Himalayan orogeny or ecological niche conservatism in karst habitats. This pattern challenges the hypothesis of strict geographic structuring proposed by Monro et al. [33]. Thus, chloroplast genome phylogeny reveals complex evolutionary histories masked by previous analyses using limited molecular markers.

### 3.4. Research Limitations and Future Directions

This study has several limitations. First, sampling covers approximately 70% of *Lysionotus* species, lacking representation of Southeast Asian endemics (e.g., Vietnamese taxa), which may constrain genus-wide evolutionary inferences. Second, the functional associations between identified hypervariable regions and adaptive traits such as epiphytism remain unexplored. Third, potential conflicts between nuclear and plastid phylogenies, influenced by processes like polyploidy and hybridization, were not addressed, although these phenomena could bias chloroplast-based phylogenies. Future research should aim to (1) sequence the remaining species to complete genus-wide plastome diversity, (2) integrate transcriptomic data to elucidate functional links between hypervariable regions and environmental adaptation, and (3) incorporate nuclear genomic data to resolve reticulate evolution, particularly in locally distributed clades with complex biogeographic histories.

### 3.5. Novel Contributions and Conservation Implications

This work represents a pioneering large-scale comparative plastome analysis within *Lysionotus*, revealing distinct patterns of genomic stability and variation between the two major clades. The identification of hypervariable regions such as *trnH-psbA* provides valuable molecular markers for species identification and germplasm screening. Moreover, our phylogenetic findings offer a molecular framework to guide conservation prioritization, exemplified by the recognition of independent evolutionary lineages in the Himalayan biodiversity hotspot (e.g., *L. atropurpureus* and *L. metuoensis*). By linking genomic variation to ecological distribution patterns, this study provides methodological insights for adaptive evolution research in Gesneriaceae and lays a theoretical foundation for the sustainable utilization of this ornamental and medicinally important genus.

## 4. Materials and Methods

### 4.1. Plant Materials

In this study, a total of 11 samples from a widely distributed clade and 13 samples from a locally distributed clade were compared, representing a total of 24 chloroplast genomes from the *Lysionotus* species. The sampling strategy was mainly based on the phylogenetic relationship obtained by Huang [43] based on nuclear ITS and chloroplast *trnl-F*, further combined with the actual situation of scientific investigation. The widely distributed clade contained *L. heterophyllus* GX, *L. aeschynanthoides*, *L. wilsonii*, *L. kwangsiensis*, *L. pauciflorus* FJ, *L. pterocaulis*, *L. pauciflorus* YN, *L. heterophyllus* GZ, *L. heterophyllus* var. *mollis*, *L. pauciflorus* var. *ikedae*, *L. microphyllus* var. *omeiensis*, both samples for *L. heterophyllus* and *L. pauciflorus*. The locally distributed clade contained *L. pubescens*, *L. atropurpureus*, *L. sessilifolius*, *L. forrestii*, *L. levipes*, *L. chatungii*, *L. guiliangii*, *L. petelotii*, *L. sulphureoides*, *L. serratus*, *L. metuoensis*, *L. gamosepalus*, also including one *Lysionotus* sp. from Motuo China. Table 1 indicates detailed information for newly sequenced plants. Voucher specimens of each collected species were deposited at Herbarium of Guangxi Institute of Botany (IBK) Guangxi Institute of Chinese Academy of Science. They were identified by Professor Weibin Xu.

### 4.2. DNA Extraction and Sequencing

Fresh samples were collected from natural habitat sites and planted in the germplasm garden of Guangxi Institute of Botany. Our collection of plant materials complied with institutional, national and international guidelines. The tissues were immediately preserved in discolored silicone and total DNA was extracted with the NuClean Plant Genomic DNA Kit from CWBIO. The quantity was evaluated by 1.0% (*w*/*v*) agarose gel electrophoresis SYBR Green I and Nanodrop2000 (Thermo Fisher Scientific, Waltham, MA, USA) to assess the concentration and purity of the DNA samples. Then, the DNA library was constructed at 250 to 300 bp, with DNA fragments converted into Illumina high-throughput sequencer-compatible forms. The high-throughput sequencing was performed on the Illumina Nova6000 Platform (Illumina, San Diego, CA, USA) and produced a total of 6 Gb of raw data each sample.

### 4.3. Plastome Genome Assembly and Annotation

The raw data were assembled under Linux using GetOrganellev1.7.5 [44], the parameters were set to get_organelle_from_reads. py-1 G_R1_001.fastq.gz-2 G_R2_001.fastq.gz -o G_output -R 45 -k 21,33,45,55,65,73,85,105,125 -f embplant_pt -t 10. The starting point of the chloroplast genomes was adjusted using seqkit [45] (version 0.12.1), and the depth of coverage was detected using bwa [46] (version 0.7.17) and samtools [47] (version 1.17) software. Sequence mapping analysis and Bandage_Windows [48] (version 0.8.1) was employed to judge the assembly quality. All 24 chloroplast genomes were assembled to obtain complete chloroplast rings. Then, the appropriate assembly fasta files were selected using Gepard [49] (version 1.40). The CPGAVAS2 [50] (version 2.1.0) and Geseq [51] (version 2.0.0) were combined to annotate the chloroplast genome, and the annotated results were manually adjusted to refer to NC_082151.1. Finally, the chloroplast genome was mapped by OGDRAW [52] (version 1.3.1).

### 4.4. Genome Comparison

Use CPJSdraw [53] (version 1.0.0) to visualize the junction boundary of quadripartite regions of the chloroplast genome, and compare the structure of the chloroplast genome.

To compare genome structures and sequence similarities among the 24 complete Gesneriaceae plastomes, sequences were aligned by Shuffle-LAGAN64 [54,55,56,57] (version 2.0.0), which could detect rearrangement, and analyze using mVISTA “http://genome.lbl.gov/vista/mvista/submit.shtml (accessed on 20 September 2023)”.

To quantify genetic diversity concretely, sequence polymorphism analysis of the 24 chloroplast genomes was performed on Dna SP [58] (version 6.15.03). A sliding window was performed every 500 bp, and one Pi was calculated every 500 bp. That is, 500 bp window length and 500 bp step size were set as the sliding window.

### 4.5. Repeats Analysis

MISA “https://webblast.ipk-gatersleben.de/misa/ (accessed on 20 September 2023)” was used to detect the chloroplast SSRs in 24 *Lysionotus* chloroplast genome sequences. The minimum numbers of repeats for mononucleotide, dinucleotides, trinucleotides, tetranucleotides, pentanucleotide, and hexanucleotides were 10, 5, 4, 3, 3, and 3, respectively [59,60,61].

REPuter “https://bibiserv.cebitec.uni-bielefeld.de/reputer/ (accessed on 20 September 2023)” was performed to identify large repeat sequences, reaching the following four repeat categories: forward, reverse, complement and palindromic repeats. The parameters were as follows: (1) setting Hamming distance as 3, (2) maximum computed repeats as 5000, and (3) minimal repeat size as 30.

### 4.6. Phylogenetic Analysis

In order to deeply understand the evolutionary relationship between the widely distributed clade and the locally distributed clade, 72 common single-copy CDs in 24 complete chloroplast genomes were extracted in PhyloSuite [62] (version 1.2.2) and performed for phylogenetic analysis. Each extracted gene was aligned with MAFFT [63] (version 7.526) and ModelFinder [64] (version 1.6.8) software was used to identify the best-fit nucleotide substitution model for the matrix based on Akaike information criterion (AIC). Maximum likelihood phylogenetices were inferred using IQ-TREE [65] (version 2.3.0) under GTR+F+I model for 1000 ultrafast bootstraps. Bayesian inference phylogenetices were inferred using MrBayes [66] (version 3.2.6), assuming different probabilities of substitution between each pair of nucleotides and accounting for rate heterogeneity among sites, i.e., the substitution rates of all sites were not equal or did not follow the same distribution. Setting Statefreqpr to Fixed (Empirical), and the Markov Chain Monte Carlo (MCMC) algorithm was employed to generate posterior probability distribution and the phylogenetic tree. The iterations number of the MCMC chain algorithm was 200,000, the output information was printed every 1000 iterations, and one sample was extracted from the MCMC chain every 1002 iterations. Two parallel sets were run in MCMC chains for Bayesian analysis, with checkpoints being saved every 5000 iterations. There were four MCMC chains in each run: one was a hot chain and the other three were cold chains, each chain starting from a different initial point, and samples were exchanged to avoid falling into local optimality, improving MCMC sampling efficiency and convergence. We summarized the MCMC sampling results using relative burn-in, discarding 27% samples, and resampling trees and parameters after the log probability stabilized, to avoid influence of the initial state on the posterior probability distribution. We evaluated all parameters in the model based on convergence diagnostics provided by the ‘sump’ and ‘sumt’ commands. Finally, we assessed the consistency and stability between different runs using the estimated effective sample size (ESS) and the potential scale reduction factor (PSRF).

### 4.7. Positive Selected Analysis

For the 24 species, single-copy CDS sequences were aligned by projecting onto corresponding amino acid alignments generated using MUSCLE [67] (version 3.8.425). The alignments were systematically evaluated for gap frequencies to assess data quality. Corresponding DNA codon alignments were subsequently refined using TRIMAL [68] (version 1.4.0) to generate high-quality, reliable alignments suitable for downstream positive selection analyses. To detect positive selection acting on individual codons along the specified lineages of *U. rockii* and *U. henryi*, the optimized branch-site model in the CODEML program of PAML [69] (version 4.10.7) was employed. Selective pressure was quantified by the nonsynonymous (dN)/synonymous (dS) substitution rate ratio (ω), where ω > 1 indicates positive selection, ω = 1 denotes neutral evolution, and ω < 1 suggests purifying selection [70]. Log-likelihood values were calculated under an alternative branch-site model (Model = 2; NSsites = 2; Fix = 0), allowing ω to vary among codons along target branches, and a null model (Model = 2; NSsites = 2; Fix = 1; Fixω = 1), enforcing neutral evolution (ω = 1) at all sites. Likelihood ratio tests (LRTs) were performed by comparing these models, with statistical significance assessed via a one-degree-of-freedom right-tailed chi-square test. *p*-values were adjusted for multiple comparisons [71]. Genes with adjusted *p*-values < 0.05 and identified positively selected sites were designated as positively selected genes (PSGs). The Bayesian Empirical Bayes (BEB) approach was applied to estimate posterior probabilities of site classes for pinpointing specific codon sites under positive selection. Codon sites with high posterior probabilities were identified as positively selected sites [38].

## 5. Conclusions

This is the first report comparing the complete chloroplast genome between 11 widely distributed and 13 locally distributed *Lysionotus* species. In this study, all 24 chloroplast genomes had typical quadripartite structure, and the number of CDs, rRNA and tRNA varied at the individual level, but were similar between the two clades. Species in the widely distributed clade had a broader length variation range in genome, LSC, SSC and IR boundary gene, while it was poorer in repeat sequence type and genetic diversity, indicating they had simple genetic variation were even more vulnerable to adverse factors. Species in the locally distributed clade owned richer repeat sequence types and higher genetic diversity. Phylogenetic analysis showed species in the locally distributed clade could form two further groups.

## Figures and Tables

**Table 1:** Taxa information of transcriptomes in this study.

Species	Sample Name	Voucher Specimen	Sample Location	Genbank Accession
*Lysionotus pauciflorus*	*L. pauciflorus* FJ	14217	Nanping, Fujian	PQ433130
*Lysionotus pauciflorus*	*L. pauciflorus* YN	14073	Funing, Yunnan	PQ468987
*Lysionotus pauciflorus* var. *ikedae*	*L. pauciflorus* var. *ikedae*	GCCC	Lanyu, Taiwan	PQ468992
*Lysionotus aeschynanthoides*	*L. aeschynanthoides*	15256	Napo, Guangxi	PQ468968
*Lysionotus pterocaulis*	*L. pterocaulis*	14228	Maguan, Yunnan	PQ468977
*Lysionotus heterophyllus*	*L. heterophyllus* GZ	HMQ1814	Panzhou, Guizhou	PQ468988
*Lysionotus wilsonii*	*L. wilsonii*	14206	Emeishan, Sichuan	PQ468969
*Lysionotus microphyllus* var. *omeiensis*	*L. microphyllus* var. *omeiensis*	WF180921	Jinfoshan, Chongqing	PQ468994
*Lysionotus heterophyllus*	*L. heterophyllus* GX	s.n.	Leye, Guangxi	PQ468967
*Lysionotus kwangsiensis*	*L. kwangsiensis*	JWS	Rongshui, Guangxi	PQ468975
*Lysionotus heterophyllus* var. *mollis*	*L. heterophyllus* var. *mollis*	GCCC	Rongshui, Guangxi	PQ468990
*Lysionotus gamosepalus*	*L. gamosepalus*	HMQ	Motuo, Tibet	PQ468978
*Lysionotus pubescens*	*L. pubescens*	14185	Tengchong, Yunnan	PQ468973
*Lysionotus atropurpureus*	*atropurpureus*	HMQ	Motuo, Tibet	PQ468986
*Lysionotus sessilifolius*	*L. sessilifolius*	14190	Gongshan, Yunnan	PQ468971
*Lysionotus forrestii*	*L. forrestii*	ZX16177	Gongshan, Yunnan	PQ468993
*Lysionotus levipes*	*L. levipes*	GCCC	Bomi, Tibet	PQ468989
*Lysionotus chatungii*	*L. chatungii*	GCCC	southeast Tibet	PQ468991
*Lysionotus guiliangii* ined.	*L. guiliangii*	2020136	Pingbian, Yunnan	PQ468983
*Lysionotus* sp.	*L.* sp.	HMQ	Motuo, Tibet	PQ468996
*Lysionotus petelotii*	*L. petelotii*	ZCY313	Malipo, Yunnan	PQ468970
*Lysionotus sulphureoides*	*L. sulphureoides*	14184	Baoshan, Yunnan	PQ468972
*Lysionotus serratus*	*L.serratus*	JZS	Longlin, Guangxi	PQ468976
*Lysionotus metuoensis*	*L. metuoensis*	HMQ	Motuo, Tibet	PQ468974

Note: ined., the abbreviation for *ineditus*, referring to unpublished species.

**Table 2:** Basic features of 24 *Lysionotus* chloroplast genomes.

Species	LSC		SSC		IR		Total		CDs	rRNA	tRNA
	Length	GC Content	Length	GC Content	Length	GC Content	Length	GC Content			
*L. pauciflorus* FJ	85,057	0.3535	17,899	0.3124	25,475	0.4317	153,906	0.3746	80	4	36
*L. pauciflorus* YN	85,131	0.3531	17,886	0.3125	25,485	0.4315	153,987	0.3743	80	4	36
*L. pauciflorus* var. *ikedae*	85,075	0.3532	17,876	0.3120	25,461	0.4314	153,873	0.3743	80	4	36
*L. aeschynanthoides*	85,078	0.3533	17,894	0.3124	25,468	0.4317	153,908	0.3745	80	4	36
*L. pterocaulis*	84,077	0.3539	17,913	0.3121	25,469	0.4316	152,928	0.3749	80	4	36
*L. heterophyllus* GZ	84,941	0.3537	17,899	0.3118	25,470	0.4318	153,780	0.3747	80	4	36
*L. wilsonii*	84,951	0.3538	17,918	0.3124	25,470	0.4318	153,809	0.3748	80	4	36
*L. microphyllus* var. *omeiensis*	84,936	0.3539	17,918	0.3124	25,471	0.4319	153,796	0.3749	80	4	36
*L. heterophyllus* GX	84,908	0.3539	17,922	0.3117	25,473	0.4318	153,776	0.3748	79	4	36
*L. kwangsiensis*	84,996	0.3536	17,746	0.3125	25,475	0.4316	153,692	0.3747	80	4	36
*L. heterophyllus* var. *mollis*	85,013	0.3535	17,747	0.3125	25,475	0.4316	153,710	0.3747	80	4	36
*L. gamosepalus*	84,944	0.3535	17,873	0.3110	25,488	0.4317	153,793	0.3745	80	4	36
*L. pubescens*	84,735	0.3532	17,854	0.3123	25,473	0.4316	153,535	0.3745	80	4	36
*L. atropurpureus*	84,592	0.3538	17,896	0.3123	25,474	0.4316	153,436	0.3748	80	4	36
*L. sessilifolius*	85,066	0.3535	17,894	0.3117	25,476	0.4319	153,912	0.3746	80	4	36
*L. forrestii*	85,069	0.3535	17,895	0.3117	25,476	0.4318	153,916	0.3746	80	4	36
*L. levipes*	84,802	0.3537	17,888	0.3128	25,477	0.4316	153,644	0.3748	80	4	36
*L. chatungii*	85,052	0.3531	17,892	0.3127	25,477	0.4316	153,898	0.3744	80	4	36
*L. guiliangii*	84,955	0.3533	17,780	0.3105	25,478	0.4317	153,791	0.3743	80	4	36
*L.* sp.	84,830	0.3537	17,887	0.3129	25,478	0.4316	153,673	0.3748	80	4	36
*L. petelotii*	84,969	0.3535	17,895	0.3098	25,478	0.4317	153,820	0.3743	80	4	36
*L. sulphureoides*	84,696	0.3533	17,892	0.3119	25,479	0.4317	153,546	0.3745	79	4	36
*L. serratus*	84,994	0.3534	17,875	0.3112	25,482	0.4318	153,833	0.3745	80	4	36
*L. metuoensis*	84,936	0.3539	17,918	0.3124	25,471	0.4319	153,796	0.3749	80	4	36

**Table 3:** The potential positive selection test based on the branch-site model.

Gene Name	Null Hypothesis			Alternative Hypothesis			Significance Test		
	lnL	df	Omega (ω = 1)	lnL	df	Omega (ω > 1)	BEB	NEB	*p*-Value
*accD*	−2248.632758	47	1	−2248.632741	48	1	NA	NA	9.95 × 10^−1^
*atpA*	−2058.307032	47	1	−2058.307032	48	1	191, N, 0.525	NA	1.00 × 10^0^
*atpB*	−2105.775158	47	1	−2105.775222	48	1	NA	NA	9.91 × 10^−1^
*atpE*	−533.527045	47	1	−533.527046	48	3.11641	NA	NA	9.99 × 10^−1^
*atpF*	−801.456315	47	1	−801.456315	48	2.89696	NA	NA	1.00 × 10^0^
*atpH*	−332.181884	47	1	−332.181918	48	1	NA	NA	9.93 × 10^−1^
*atpI*	−1009.601571	47	1	−1009.601571	48	1.43147	NA	NA	1.00 × 10^0^
*cemA*	−987.48884	47	1	−987.376114	48	999	73, L, 0.550	NA	6.35 × 10^−1^
*clpP*	−866.539298	47	1	−866.539298	48	1	56, N, 0.762/73, M, 0.763	NA	1.00 × 10^0^
*infA*	−324.952445	47	1	−324.952445	48	3.36607	NA	NA	1.00 × 10^0^
*matK*	−2289.050163	47	1	−2289.040516	48	62.13547	NA	NA	8.90 × 10^−1^
*ndhA*	−1615.94473	47	1	−1615.71938	48	74.33031	188, S, 0.945	NA	5.02 × 10^−1^
*ndhB*	−2056.657197	47	1	−2056.425012	48	455555	300, T, 0.760	NA	4.96 × 10^−1^
*ndhC*	−470.93447	47	1	−470.691545	48	2.537453	30, F, 0.723	NA	4.86 × 10^−1^
*ndhD*	−1657.695752	47	1	−1657.695665	48	5.37607	NA	NA	9.89 × 10^−1^
*ndhE*	−797.350973	47	1	−796.338806	48	1	6, V, 0.667/92, G, 0.667	NA	1.55 × 10^−1^
*ndhF*	−3479.498086	47	1	−3479.495536	48	268.69832	NA	NA	9.43 × 10^−1^
*ndhG*	−745.874227	47	1	−745.874237	48	3.45598	NA	NA	9.96 × 10^−1^
*ndhH*	−1658.650947	47	1	−1658.544271	48	183.30161	321, L, 0.652	NA	6.44 × 10^−1^
*ndhI*	−686.641873	47	1	−686.641873	48	1	NA	NA	1.00 × 10^0^
*ndhJ*	−648.355572	47	1	−648.355572	48	3.96959	NA	NA	1.00 × 10^0^
*ndhK*	−928.671632	47	1	−928.671632	48	2.73846	NA	NA	1.00 × 10^0^
*petA*	−1410.28301	47	1	−1410.282903	48	12.57637	NA	NA	9.88 × 10^−1^
*petB*	−648.640314	47	1	−648.640433	48	3.49232	NA	NA	9.88 × 10^−1^
*petD*	−636.531041	47	1	−636.531041	48	1.73442	NA	NA	1.00 × 10^0^
*petG*	−144.952513	47	1	−144.952513	48	3.96269	NA	NA	1.00 × 10^0^
*petL*	−110.86864	47	1	−110.868657	48	1.0009	NA	NA	9.95 × 10^−1^
*petN*	−110.133659	47	1	−110.133721	48	4.82041	NA	NA	9.91 × 10^−1^
*psaA*	−3206.269402	47	1	−3206.269403	48	2.98077	NA	NA	9.99 × 10^−1^
*psaB*	−3062.739322	47	1	−3062.738803	48	3.12016	NA	NA	9.74 × 10^−1^
*psaC*	−323.719242	47	1	−323.719242	48	4.44198	NA	NA	1.00 × 10^0^
*psaI*	−131.283698	47	1	−131.283628	48	2.33483	NA	NA	9.91 × 10^−1^
*psaJ*	−179.165779	47	1	−179.165779	48	3.35833	NA	NA	1.00 × 10^0^
*psbA*	−1466.554562	47	1	−1466.554562	48	3.87052	NA	NA	1.00 × 10^0^
*psbB*	−2109.542106	47	1	−2109.536245	48	2.43776	NA	NA	9.14 × 10^−1^
*psbC*	−1953.325342	47	1	−1953.224272	48	2.594515	NA	NA	6.53 × 10^−1^
*psbE*	−326.594616	47	1	−326.593561	48	1	NA	NA	9.63 × 10^−1^
*psbF*	−160.673626	47	1	−160.673626	48	3.75946	NA	NA	1.00 × 10^0^
*psbH*	−287.330874	47	1	−287.330874	48	1	NA	NA	1.00 × 10^0^
*psbI*	−142.497656	47	1	−142.497705	48	3.67788	NA	NA	9.92 × 10^−1^
*psbJ*	−142.497656	47	1	−154.654	48	3.6036	NA	NA	9.98 × 10^−1^
*psbK*	−217.948136	47	1	−217.948136	48	3.03544	NA	NA	1.00 × 10^0^
*psbL*	−154.805662	47	1	−154.805678	48	3.42968	NA	NA	9.95 × 10^−1^
*psbM*	−129.554891	47	1	−129.554864	48	1.61771	NA	NA	9.94 × 10^−1^
*psbN*	−171.012236	47	1	−171.011114	48	1	NA	NA	9.62 × 10^−1^
*psbT*	−134.337509	47	1	−134.337528	48	1	NA	NA	9.95 × 10^−1^
*rbcL*	−2076.746457	47	1	−2071.211324	48	3.145512	NA	NA	8.77 × 10^−4^
*rpl14*	−483.945572	47	1	−483.945572	48	3.13388	NA	NA	1.00 × 10^0^
*rpl16*	−416.903274	47	1	−416.903274	48	3.56892	NA	NA	1.00 × 10^0^
*rpl2*	−1103.495182	47	1	−1103.495439	48	3.32559	NA	NA	9.82 × 10^−1^
*rpl20*	−568.82109	47	1	−568.604892	48	43.34374	NA	NA	5.11 × 10^−1^
*rpl22*	−523.228276	47	1	−523.040286	48	41.52047	NA	NA	5.40 × 10^−1^
*rpl23*	−363.661484	47	1	−363.660276	48	5.72649	NA	NA	9.61 × 10^−1^
*rpl32*	−249.962411	47	1	−249.957138	48	3.364021	NA	NA	9.18 × 10^−1^
*rpl33*	−282.055455	47	1	−282.055454	48	3.49801	NA	NA	9.99 × 10^−1^
*rpl36*	−147.104844	47	1	−147.104845	48	3.68001	NA	NA	9.99 × 10^−1^
*rpoA*	−1644.775529	47	1	−1644.372116	48	73.59795	NA	NA	3.69 × 10^−1^
*rpoB*	−4556.139932	47	1	−4556.139933	48	3.54957	NA	NA	9.99 × 10^−1^
*rps11*	−607.498798	47	1	−607.498786	48	1	NA	NA	9.96 × 10^−1^
*rps14*	−462.578581	47	1	−462.578586	48	3.43329	NA	NA	9.97 × 10^−1^
*rps15*	−425.650605	47	1	−425.650263	48	2.96167	NA	NA	9.79 × 10^−1^
*rps16*	−301.101204	47	1	−301.101204	48	3.32542	NA	NA	1.00 × 10^0^
*rps18*	−301.10126	47	1	−301.101204	48	3.73522	NA	NA	9.92 × 10^−1^
*rps2*	−1060.616195	47	1	−1060.616195	48	1	NA	NA	1.00 × 10^0^
*rps3*	−972.616253	47	1	−972.616106	48	3.28734	NA	NA	9.86 × 10^−1^
*rps4*	−858.00646	47	1	−858.027768	48	3.12	NA	NA	8.36 × 10^−1^
*rps7*	−601.835587	47	1	−601.836044	48	3.28237	NA	NA	9.76 × 10^−1^
*rps8*	−615.141664	47	1	−614.894166	48	3.489105	24, V, 0.840	NA	4.82 × 10^−1^
*ycf1*	−1433.829235	47	1	−1433.570645	48	83.60908	11, L, 0.510	NA	4.72 × 10^−1^

## Data Availability

The data generated in this study are available at Genbank (accession numbers: PQ468967-PQ468996 and PQ433130).

## Supplementary Materials

The following supporting information can be downloaded at https://www.mdpi.com/article/10.3390/ijms26157031/s1.
