# Supplementary material for: Comparative Analysis of Chloroplast Genome Between Widely Distributed and Locally Distributed Lysionotus (Gesneriaceae) Related Members
Source: Int J Mol Sci. 2025 Jul 22;26(15):7031. doi: 10.3390/ijms26157031 (PMC12345977; doi:10.3390/ijms26157031)
Supplement: Supplementary file 1 [file Figure S1. Comparison and analysis based on chloroplast genome of 24 samples.pdf › Figure S1. Comparison and analysis based on chloroplast genome of 24 samples.pdf]

Lysionotus pauciflorus FJ:1-153906

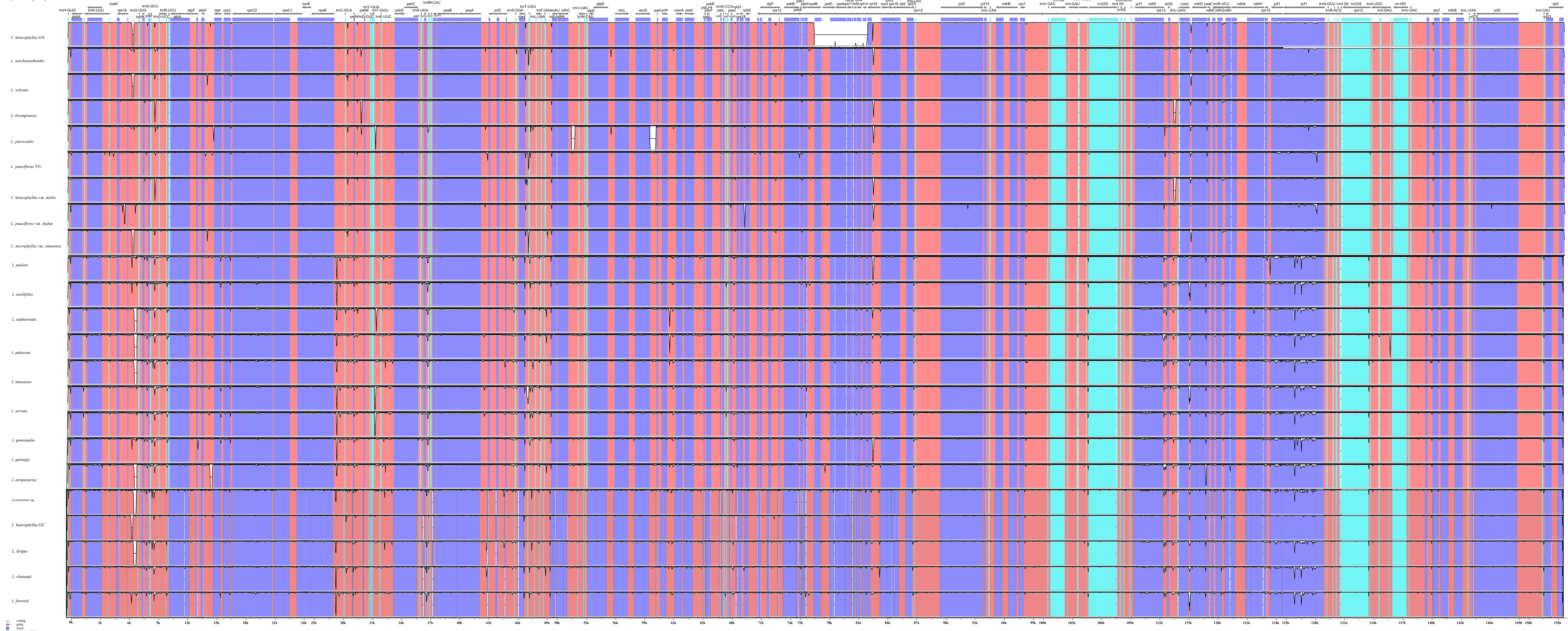

S1.Comparison and analysis based on chloroplast genome of 24 samples. Orientation of genes were pointed out by arrows up the alignments. Purple, blue, pink and grey bars correspond to exons, untranslated regions, non-coding sequences and mRNA respectively. Y-axis indicates the genetic similarity percentage. Genetic similarity among 50-100% were shown in the figure.
